# The Late Meeting of the Georgia Medical Association

**Published:** 1899-05

**Authors:** 


### EDITORIAL NOTES AND COMMENTS.

The Business office of THE JOURNAL-RECORD is 305, 306 Fitten Building.

The Editorial office is Rooms 401, 402 Grand Opera House.

Address all Business communications to Dr. M. B. Hutchins, Mgr.

Make remittances payable to THE ATLANTA JOURNAL-RECORD OF MEDICINE.

On matters pertaining to the Editorial and Original Communications address Dr. Dunbar Roy, Grand Opera House, Atlanta.

Reprints of original articles will be furnished at cost price. Requests for the same should always be made *on the manuscript*.

We will present, post-paid, on request, to each contributor of an original article, twenty (20) marked copies of THE JOURNAL-RECORD containing such article.

### THE LATE MEETING OF THE GEORGIA MEDICAL ASSOCIATION.

THE fiftieth annual meeting of the State Medical Association was held in Macon on April 19, 20, 21. It was a semi-centennial celebration, and proved a great success. There was the largest attendance ever known in the history of the Association, and the program of papers showed an excellency throughout the list. Much of the success of the meeting was due to the untiring efforts of its President, Dr. Howard Williams, who began working soon after his election at Cumberland last year, and never ceased until the last paper of the program had been read. The program consisted of sixty-three papers, embracing almost all subjects in the domain of medicine, and, besides, there were four addresses delivered by distinguished men from a distance.

Dr. Hare, of Philadelphia, who sent a telegram at the last moment saying that he was unavoidably detained, presented a most excellent address, which was read by Dr. C. D. Hurt, on "Some

Facts in Regard to the Medical Complications, Sequela, and Treatment of Typhoid Fever."

Dr. Senn also sent his address on "Emergency Surgery." Both of these addrsses will be published in full in this JOURNAL.

Dr. Jos. Price, of Philadelphia, was present, and contributed to the program by making an address on "An Analysis of Appendicitis Operative Methods."

Dr. Jas. P. Tuttle, of New York, contributed an address on "Rectal Surgery."

Taking it altogether, the program was excellent, and some of the papers brought forth interesting discussions.

Notably must be mentioned the paper of Dr. T. V. Hubbard, of Atlanta, on "The Eliminative or Woodbridge Treatment of Typhoid Fever."

The Symposium on Ether Anesthesia was greatly enjoyed, and the papers on the subject were excellent.

The social features of the meeting were all that could be desired. Macon is especially known for her munificence in this direction, and on this occasion nothing was left undone. On the first evening a concert was given at the Blind Asylum complimentary to the visiting physicians, and at 9 o'clock the Chamber of Commerce entertained all the members of the Association at an informal "Bohemian Smoker" in the Elk's Hall. This proved to be especially enjoyable, and much success was due to its President, who exerted himself in every way to make the visitors feel at home. Good jokes were told, good cigars smoked, and there was a feeling of general good cheer. Mr. Harry Edwards, the gifted writer, entertained a large circle of guests with one of his witty, humerous talks. On Tuesday and Friday, from 12 to 2, the Acme Brewing Co., of Macon, served a luncheon, and incidentally some of their most excellent beer. Some of our Augusta friends tell us that the beer was excellent.

On Thursday evening the crowning event of the occasion was given at the Progress Club, in the form of a reception. Music and dancing, mingled with the odor of sweet flowers, "made the young hearts grow younger. Fair matrons and beautiful belles of Macon added much to making the occasion one long to be remembered.

The Association adjourned on Friday to meet next year in Atlanta. We only hope that we may be able to imitate Macon.

The officers elected were the following:

President—Dr. F. W. McRae, of Atlanta.

First Vice-President—Dr. J. B. Graham, of Savannah.

Second Vice-President—Dr. H. B. McMaster, of Waynesboro.

Secretary—Dr. R. L. Taylor, of Griffin.

Treasurer—Dr. E. C. Goodrich, of Augusta.

